# Synthesis Approaches to (−)-Cytoxazone, a Novel Cytokine Modulator, and Related Structures

**DOI:** 10.3390/molecules21091176

**Published:** 2016-09-06

**Authors:** Izabel L. Miranda, Ítala K. B. Lopes, Marisa A. N. Diaz, Gaspar Diaz

**Affiliations:** 1 Departamento de Química, Universidade Federal de Minas Gerais, Belo Horizonte 31270-901, Brazil; izabelmirandaqui@gmail.com (I.L.M.); italakariny@hotmail.com (Í.K.B.L.); 2 Departamento de Bioquímica e Biologia Molecular, Universidade Federal de Viçosa, Viçosa 36570-000, Brazil; marisanogueira@ufv.br

**Keywords:** (−)-cytoxazone, organic synthesis, stereoselective synthesis, biological activity, antimicrobial activities, cytokine modulator

## Abstract

(−)-Cytoxazone, originally isolated from cultures of a *Streptomyces* species has an oxazolidin-2-one 4,5-disubstituted ring. It is known that this natural product presents a cytokine modulator effect through the signaling pathway of Th2 cells (type 2 cytokines), which are involved in the process of growth and differentiation of cells. From this, the interest in the development of research aimed at the total synthesis of this molecule and its analogs has remained high, which can be confirmed by the large number of publications on the topic, more than 30 to date. This review focuses on the various creative methods for the synthesis of (−)-cytoxazone and its congeners. The assessment of the preparation of this oxazolidinone and related structures serves as a treatise on the efforts made in the synthesis of this important class of compound from its first total synthesis in 1999.

## 1. Introduction

This review focuses on the various total synthesis strategies for the preparation of (−)-cytoxazone and its congeners. The assessment of the preparation of this oxazolidinone and related structures serves as a treatise on the efforts made in the synthesis of this class of compounds. Emphasis is placed on the literature since the first total synthesis in 1999 until mid-2016. Readers may also consult two excellent surveys of the building of oxazolidin-2-one rings by Rozwadowska et al. [1] and Zappia [2]*.*

### 1.1. Compounds Containing 2-Oxazolidinone Structural Units and Biological Applications

**Figure 1:**
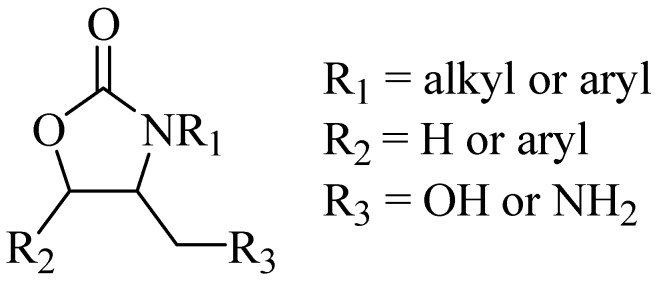
Antimicrobial oxazolidin-2-ones.

New synthetic antimicrobial agents have been discovered from the preparation of a library of compounds of the oxazolidin-2-one family (Figure 1) by DuPont researchers [3,4], specifically exerting a bacteriostatic effect during *in vitro* and *in vivo* assays, using human pathogenic bacteria [3].

The main biological activities exhibited by oxazolidin-2-ones include antiallergic, psychotropic, and neuroleptic activities. Moreover, oxazolidin-2-ones are also intermediates in the synthesis of renin inhibitors, β-lactams and macrolide antibiotics [5].

**Figure 2:**
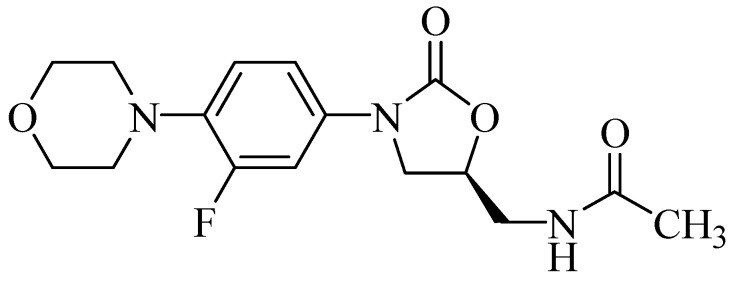
Antibacterial linezolid (PNU-100766).

This class of compounds also exhibited remarkable activities against Gram-positive bacteria such as *Staphylococcus aureus*, *Staphylococcus epidermidis*, *Streptococcus pneumoniae*, *Enterococcus faecalis*, and *Enterococcus faecium*. They are also active against pathogens that are resistant to one or more antibiotics, as is the case with methicillin-resistant *S. aureus*, vancomycin-resistant enterococci, and penicillin-resistant pneumococci [4]. By contrast, the 5-substituted oxazolidinones exhibit an even more evident antibacterial activity. Linezolid (PNU-100766, Figure 2), for example, is recommended for the treatment of the infections caused by Gram-positive bacteria, such as those caused by vancomycin-resistant *S. aureus* [6], and was the first compound of this class to be licensed in the United States by the Food and Drug Administration (FDA) in 2000 [4].

**Figure 3:**
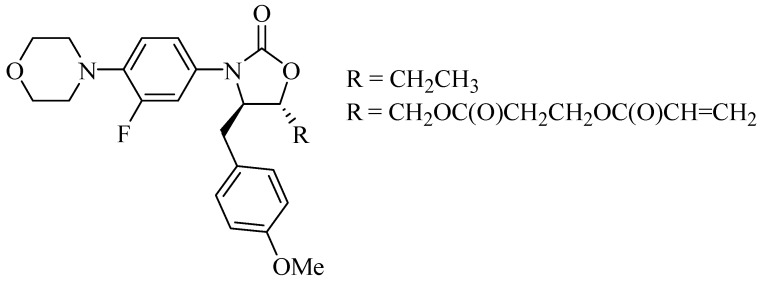
Oxazolidinone compounds 4,5-di-substituted with anticancer activity.

Furthermore, based on reports regarding new mechanisms of action against cancer, HIV, monoamine oxidase, glutamate receptor antagonists, and metabotropic receptors, Naresh et al. [7] synthesized a series of compounds that were linezolid analogs. Those, which had acrylic and ethyl substituents (Figure 3), exhibited excellent results against lung and prostate cancer cells, revealing a new option for the synthesis of drugs for the treatment of these types of cancer.

In addition to the reported range of biological activities, after Evans reported their use as chiral auxiliaries in 1981, some of these oxazolidinones have been widely used in research involving asymmetric organic syntheses [2].

### 1.2. (−)-Cytoxazone

**Figure 4:**
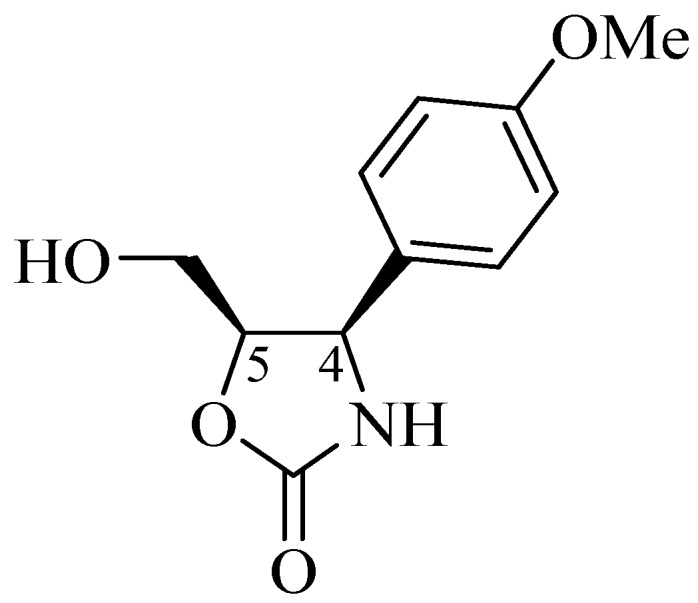
Structure of (−)-cytoxazone.

Although quite rare, a new type of oxazolidin-2-one [2], (−)-cytoxazone (Figure 4), was originally isolated by Kakeya et al. [8] from cultures of a *Streptomyces* species. (−)-Cytoxazone has an oxazolidin-2-one 4,5-disubstituted ring, and its absolute configuration, based on NMR data, CD, and X-ray crystallography, was defined as (4*R*,5*R*)-5-hydroxymethyl-4-*p*-methoxyphenyl-1,3-oxazolidin-2-one [8,9]. Nakata et al. [10] unequivocally confirmed the absolute configuration resulting from the first asymmetric total synthesis.

It is known that the induction of humoral antibodies or cellular responses are affected by different sets of cells. The set of cells called Th1 (type 1 cytokines) are responsible for hypersensitivity reactions, however, the Th2 set (type 2 cytokines) are involved in the processes of cell growth and differentiation.

An imbalance in the production of these cytokines can result in immunological disorders such as allergies, progressive lymphoproliferation and severe immunodeficiency [9], since they are directly related to the production of antibodies. Looking for new immunomodulatory chemicals able to control the production of type 2 cytokines, Kakeya et al. [9] found that (−)-cytoxazone presented a cytokine modulator effect through the signaling pathway of Th2 cells, but not of Th1 cells. From this, the interest in the development of methods for the total synthesis of (−)-cytoxazone and its analogs has remained high, which can be confirmed by the large number of publications, more than 30 to date. In this context, the following review consists of a compilation of information about the synthesis of (−)-cytoxazone from its first approaches to the present day, as it is understood that this is a valuable contribution to the study of new synthetic strategies for this oxazolidinone, a very important molecule in the therapeutic arsenal today.

## 2. Total Synthesis Strategies of (−)-Cytoxazone and Congeners

The description of all publications on the preparation of (−)-cytoxazone and some congeners are presented in chronological order starting from 1999, when the first total syntheses of (−)-cytoxazone (**1**) were published, simultaneously, by Nakata [10] and Mori [11], respectively, both starting from 4-methoxycinnamate **2**. Exhaustive coverage, however, cannot be guaranteed. 

### 2.1. Stereoselective Syntheses of (−)-Cytoxazone, a Novel Cytokine Modulator

**Scheme 1:**
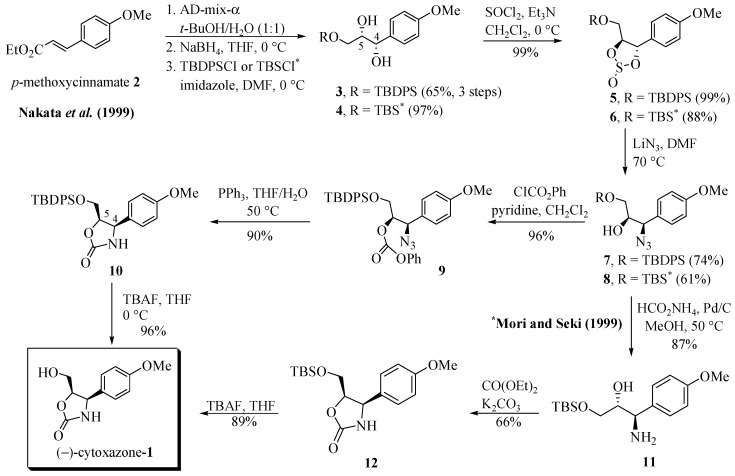
First total syntheses of (−)-cytoxazone reported by Nakata et al. [10] and Mori and Seki [11].

The route proposed by Nakata et al. [10] is summarized in Scheme 1. The key step of the synthesis of **1** consisted of a regio- and stereoselective introduction of an azide group into 4-methoxy-cinnamate **2**. The sequence of Sharpless asymmetric dihydroxylation from **2**, using AD-mix-α in *t*-BuOH/H_2_O, the reduction of the ester group with NaBH_4_, and the regioselective protection of the primary hydroxyl function with *t*-butyldiphenylsilyl chloride (TBDPSCl) afforded the (4*S*,5*S*)-diol **3** (cytoxazone numbering) in 65% yield (three steps). Treatment with SOCl_2_ and Et_3_N afforded the cyclic sulfite **5** in 99% yield. The regio- and stereoselective opening of sulfide was made by a nucleophilic reaction with LiN_3_ in DMF to generate azido alcohol **7** (74% yield) and desilylated azido-diol (24% yield) which was converted quantitatively to **7** by TBDPS/imidazole treatment. The azido alcohol **7** was treated with ClCO_2_Ph/pyridine, providing the phenyl carbonate **9** in 96% yield. The heterocyclization was performed in one pot from **9** by reduction of azide group to amine with Ph_3_P in THF/H_2_O and concomitant cyclization to produce oxazolidinone **10** (90% yield) as a properly controlled stereochemistry. Finally, the removal of the TBDPS group with TBAF, produced the desired (−)-cytoxazone (**1**) in 96% yield.

While Nakata et al. [10] converted the azido alcohol **7** into the corresponding phenyl carbonate **9**, Mori and Seki [11] converted the azido alcohol **8** into the amino alcohol **11** in 87% yield, by reducing the azide group with ammonium formate and Pd/C (Scheme 1). *N,O*-Heterocyclization of **11** was accomplished by treatment with diethyl carbonate, providing **12** in 66% yield. The desilylation of **12** produced (−)-cytoxazone in 89% yield. These two studies established the absolute stereochemical configuration of (−)-cytoxazone.

### 2.2. Chemoenzymatic Synthesis of (−) and (+)-Cytoxazone Enantiomers

**Scheme 2:**
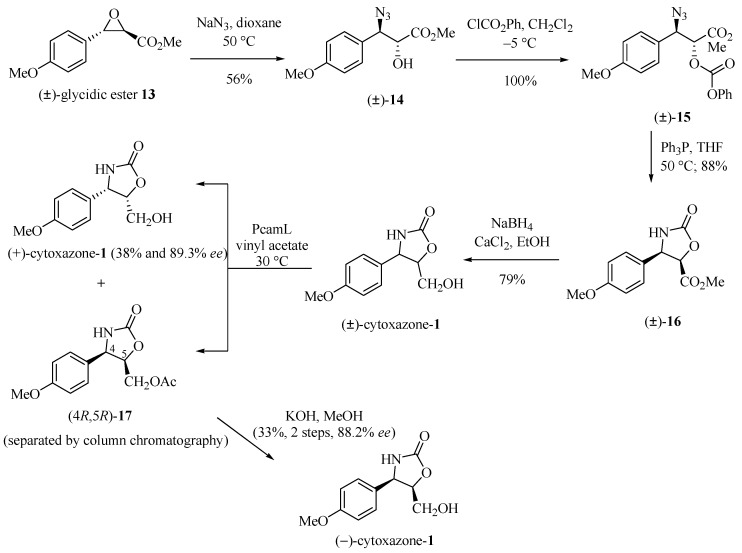
Chemoenzymatic synthesis of the (−)- and (+)-cytoxazones [12].

Sunjic and co-workers [12] described the chemoenzymatic synthesis of the enantiomers (−)- and (+)-cytoxazone whose resolution from the racemate (±)-**1** was achieved by esterification catalyzed by *Penicillium camambertii* lipase (PcamL) (Scheme 2). Preparation of (±)-**1** starts with the opening of the glycidic epoxide **13** by treatment with NaN_3_, producing azido alcohol **14** with complete regio- and stereoselectivity, in accordance with a well-stablished mechanism [13]. Treatment of this intermediate with ClCO_2_Ph produced the phenyl carbonate (±)-**15**. The reduction of the azide group and simultaneous cyclization generated (±)-oxazolidinone **16**. Finally, the reduction of the ester group with sodium borohydride yielded (±)-cytoxazone (**1**) in 79% yield. Enzymatic kinetic resolution under acetylation of (±)-**1** with vinyl acetate by PcamL-catalyzed and after their separation by chromatography column, produced the acetate (4*R*,5*R*)-**17** and the (+)-cytoxazone (38% yield and 89.3% *ee*). Hydrolysis of ester **17** with KOH in MeOH produced (−)-cytoxazone (33% yield, two steps, and 88.2% *ee*).

**Scheme 3:**
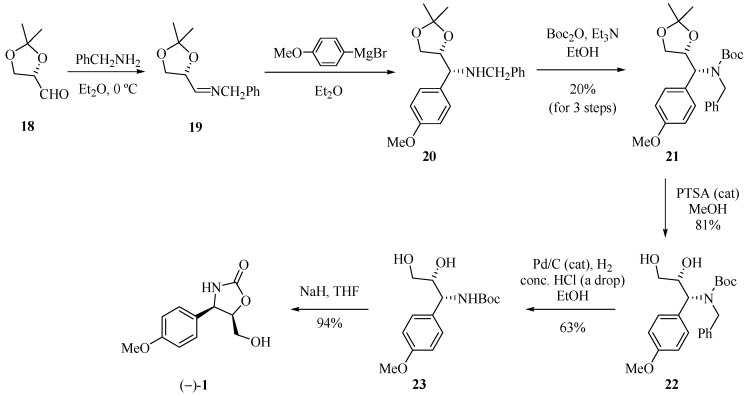
Total synthesis of (−)-cytoxazone proposed by Rao and co-workers [14].

The synthetic approach reported by Rao and his group [14] to obtain (−)-cytoxazone started from (*S*)-2,3-*O*-isopropylidene glyceraldehyde (**18**, Scheme 3). Treatment of this compound with benzylamine produced imine **19**, which when subjected to stereoselective addition of *p*-methoxyphenylmagnesium bromide led to amine **20**. The amine function was protected with Boc_2_O producing the diprotected amine **21** (20% yield, three steps). Finally, the sequence of a chemoselective hydrolysis of the isopropylidene unit, debenzylation, and regioselective cyclization gave (−)-cytoxazone (**1**) from glyceraldehyde **18** in 9.6% overall yield.

**Scheme 4:**
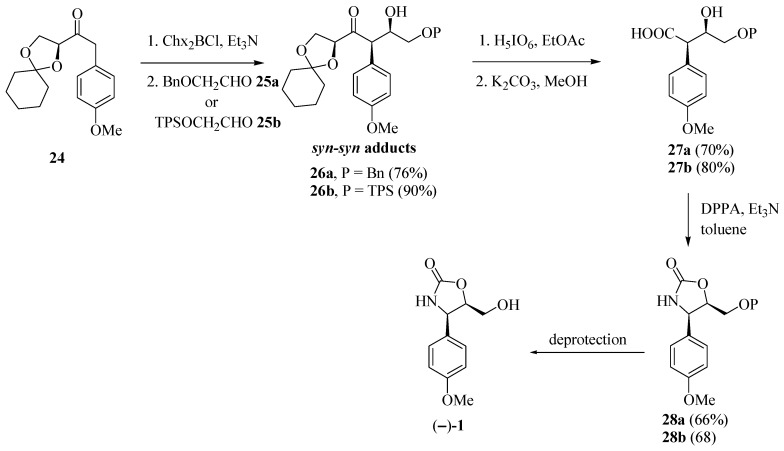
Total synthesis of (−)-cytoxazone proposed by Marco et al. [15].

Marco and co-workers [15] obtained enantiomerically pure (−)-cytoxazone by a route that included a *syn*-stereoselective aldol addition by chlorodicyclohexylborane (Chx_2_BCl) mediation, with the unusual chiral ketone **24** and a Curtius rearrangement representing the key steps (Scheme 4). The aldol addition of ketone **24** to protected glycol aldehydes **25a**–**b**, provided the expected *syn-syn* aldol adducts **26a**–**b**. Oxidative cleavage of the acetonide rings by treatment with periodic acid provided the β-formyloxy acid intermediaries, which have been converted, subsequently, in basic conditions, into hydroxy acids **27a**–**b**. Curtius rearrangement of these compounds was made by treatment with diphenyl phosphoryl azide (DPPA), whose isocyanate groups in the formed intermediates, were captured *in situ* by free hydroxyl groups, leading to the formation of oxazolidinone rings **28a**–**b**, which after *O*-deprotection, produced (−)-cytoxazone.

### 2.3. A New Synthesis of (−)-Cytoxazone and Its Diastereomers

**Scheme 5:**
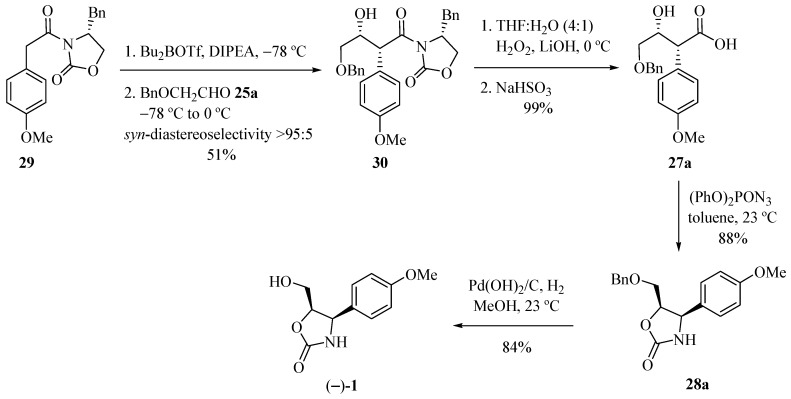
Total synthesis of (−)-cytoxazone reported by Carter and co-workers [16].

Carter and his team [16] explored a new (−)-cytoxazone synthesis route by means of an aldol reaction, starting from the chiral imide **29** with an aldehyde mediated by Bu_2_BOTf (Scheme 5). Subsequent reaction of the dibutylboron enolate of **29** with benzyloxyacetaldehyde **25a**, according to the pioneering work of Evans [17], produced aldol **30** in good *syn*-diastereoselectivity (>95:5). Removal of the chiral auxiliary from **30** by treatment with lithium hydroxyperoxide led to the carboxylic acid **27a** in 99% yield. The heterocyclization of the acid **27a** was performed in a one pot (three-step) (acyl azide formation, Curtius rearrangement, isocyanate trapping) using diphenylphosphoryl azide producing the ether-oxazolidinone **28a**. Deprotection using Pd(OH)_2_/C and hydrogen atmosphere produced enantiomerically pure (−)-cytoxazone in 84% yield. The enantiomer (+)-**1** was similarly prepared to natural cytoxazone from (*S*)-4-phenylmethyl-oxazolidin-2-one.

**Scheme 6:**
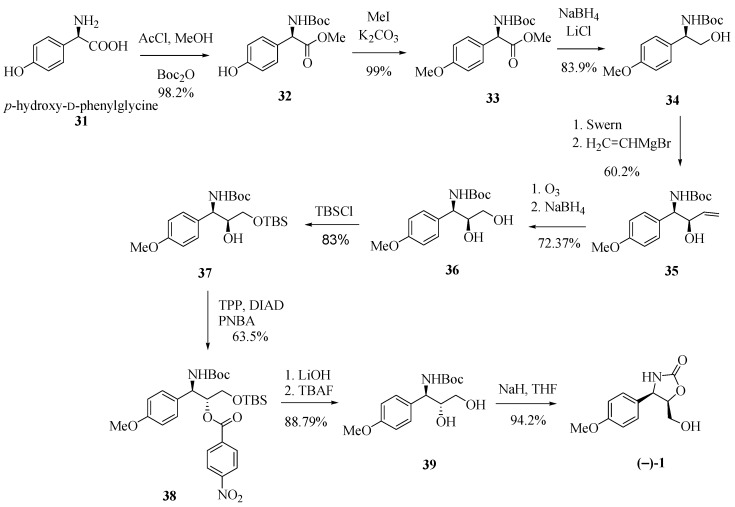
Synthesis of (−)-cytoxazone via stereoselective Grignard addition [18].

In another contribution to the preparation of (−)-cytoxazone, Rao and co-workers [18] proposed a stereoselective addition reaction of a vinyl Grignard at the *N*-Boc-aldehyde **34a**, obtained from *p*-hydroxy-D-phenylglycine (**31**, Scheme 6). Starting from **31**, the sequence of esterification, *N*-Boc-protection, phenol methylation, and the reduction of the ester function generated alcohol **34** in 81.6% yield (four steps). Swern oxidation of **34** followed by stereoselective addition of vinylmagnesium bromide to the resulting aldehyde led to the *syn*-1,2-amino alcohol **35** in 60.2% yield. Ozonolysis of the double bond and reduction of the formed aldehyde produced diol **36**. Protection of the primary hydroxyl group, followed by an inversion of the configuration of the secondary hydroxyl by means of a Mitsunobu condition and subsequent desilylation led to *trans*-1,2-amino alcohol **39** in 46.79% overall yield from **36**. Finally, the *N,O*-heterocyclization generated (−)-cytoxazone in 94.2% yield.

### 2.4. Asymmetric Synthesis of (4R,5R)-Cytoxazone

**Scheme 7:**
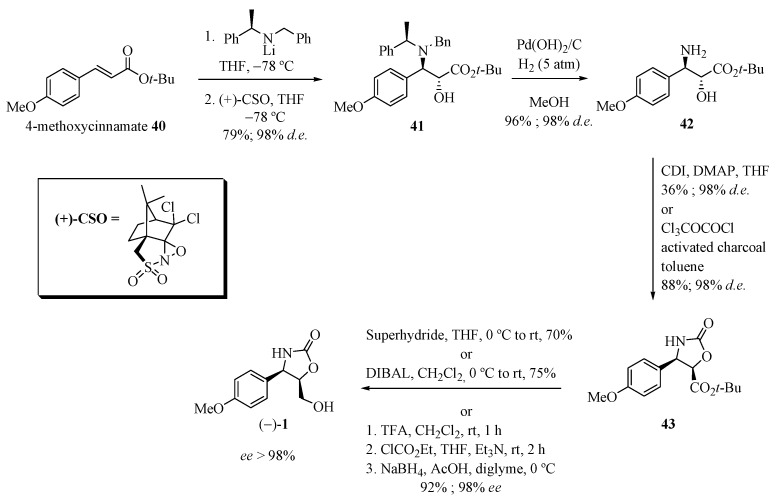
Total synthesis of (−)-cytoxazone reported by Davies and co-workers [19].

Davies and co-workers [19] reported the synthesis of (−)-cytoxazone in four steps from 4-methoxycinnamate (Scheme 7). 1,4-Addition of lithium (*R*)-*N*-benzyl-*N*-methylbenzylamide to cinnamate **40** and subsequent diastereoselective enolate oxidation using (+)-(camphorsulfonyl)-oxaziridine) [(+)-CSO] produced the α-hydroxy-β-amino ester **41**. Hydrogenolysis of the *N*-benzyl group in the presence of Pearlman’s catalyst provided the free amino alcohol **42**. Treatment with diphosgene generated **43** (88% yield and 99% *de*). Finally, the reduction of the *tert*-butyl ester group with superhydride or DIBAL-H produced (−)-cytoxazone in 70% or 75% yields, respectively, and with an enantiomeric excess of higher than 98%.

### 2.5. Short Synthesis of Both Enantiomers of Cytoxazone using the Petasis Reaction

**Scheme 8:**
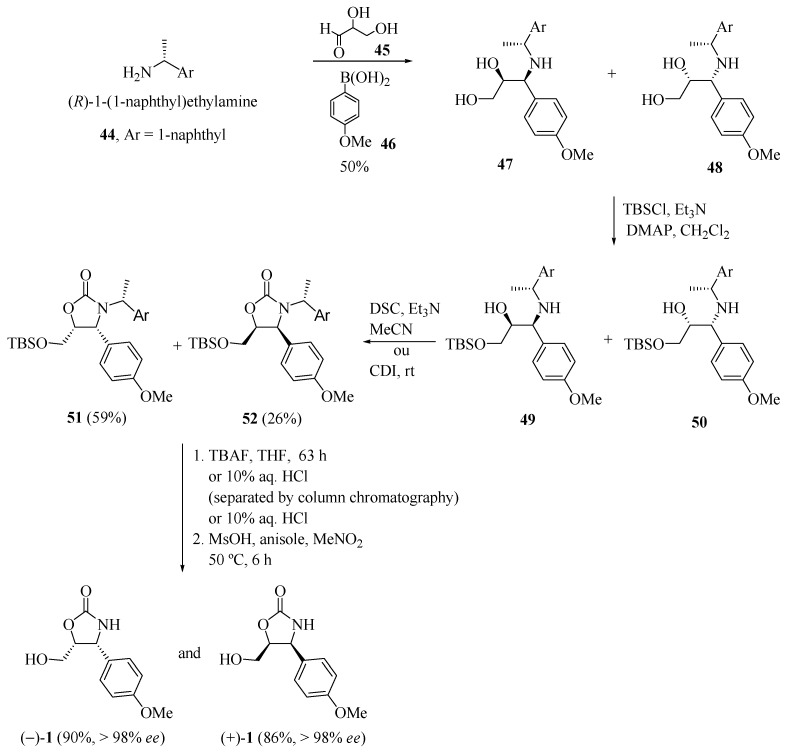
Synthesis of the enantiomers of cytoxazone through Petasis coupling [21].

Using Petasis’s protocol [20], Sugiyama and co-workers [21] reported the synthesis of (−)-cytoxazone (Scheme 8). The coupling reaction between three components, (*R*)-1-(1-naphthyl)ethylamine (**44**), d,l-glyceraldehyde (**45**) and 4-methoxyphenylboronic acid (**46**) led to a diastereomeric mixture (1:1) of the aminodiols **47** and **48** in 50% yield. Protection of the primary hydroxyl of mixture led to their silyl ethers **49** and **50**. Oxazolidin-2-one rings **51** and **52** were formed from **49** and **50** upon treatment with *N,N'*-succinimidyl carbonate (DSC) in acetonitrile. Finally, the sequence of desilylation, the separation of the diastereomeric mixture by column chromatography, and the removal of the 1-naphthylethyl group, generated both enantiomers of cytoxazone (−)-**1** and (+)-**1**.

Alternatively, the (−)- and (+)-cytoxazone enantiomers were also obtained in a one-pot process from the diastereomeric mixture of **47** and **48** (Scheme 8). The sequence of the selective protection of the primary hydroxylic groups of these diols, heterocyclization with 1,1-carbonyl *bis*-1*H*-imidazole (CDI), desilylation with 10% HCl, and the removal of the 1-naphthylethyl group led to (−)- and (+)-cytoxazone in 13% and 7% overall yields, respectively. In the one-pot process, CDI, rather than DSC, was used for the cyclization process due to its low solubility in dichloromethane, and 10% HCl, rather than TBAF, was used for desilylation to reduce the formation of unwanted products. 

### 2.6. Imino 1,2-Wittig Rearrangement of Hydroximates and Its Application to the Synthesis of (−)-Cytoxazone

**Scheme 9:**
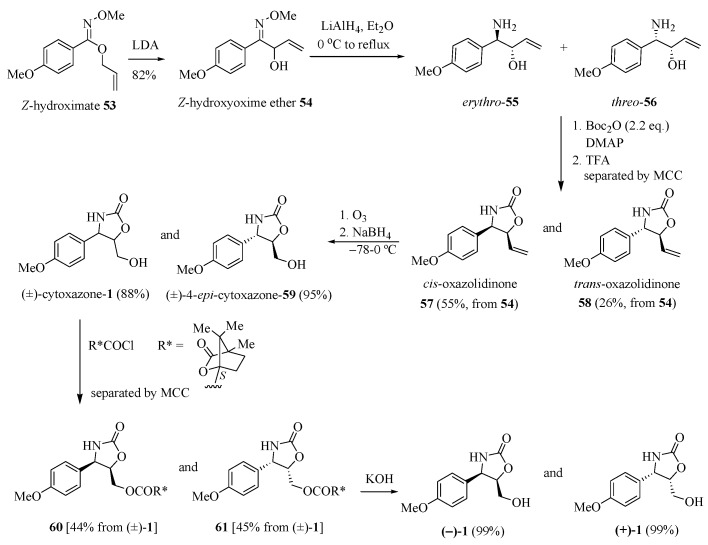
Synthetic approach of (−)- and (+)-cytoxazone reported by Naito’s group [22].

Naito and his group [22] reported a synthetic approach to prepare the (−)-cytoxazone based on the imino 1,2-Wittig rearrangement of hydroximates (Scheme 9). The *Z*-hydroximate **53**, when treated with LDA, underwent 1,2-Wittig rearrangement to give *Z*-2-hydroxyoxime ether **54**. Reduction and concomitant *N*-demethoxylation of **54** with LiAlH_4_ yielded a mixture of *erythro*- and *threo*-amino alcohols **55** and **56**, respectively. Heterocyclization in situ of the mixture through the *N*-Boc di-protection by treatment with di-*tert*-butyl dicarbonate (Boc_2_O) (2.2 equiv.) in the presence of DMAP and subsequent *N*-Boc-deprotection with TFA led, after the separation of diastereomers, to *cis*- and *trans-*oxazolidin-2-one derivatives **57** and **58** in 55% and 26% yields, respectively (three steps). These oxazolidinone derivatives were separately converted into (±)-cytoxazone (**1**) and (±)-4-*epi*-cytoxazone (**59**) through the oxidative cleavage of terminal alkene with ozone and subsequent reduction with NaBH_4_ of the formed aldehyde. The chemical resolution of the racemate (±)-**1** was carried out by acylation with (−)-camphanic chloride to give (4*R*,5*R*)- and (4*S*,5*S*)-oxazolidinones **60** and **61** in 44% and 45% yields, respectively, after the separation of isolated diastereomers by medium-pressure column chromatography (MCC). Hydrolysis of **60** and **61** led, respectively, to (−)- and (+)-**1**. Analogously, the enantiomers (−)- and (+)-4-*epi*-cytoxazone were also prepared from racemate (±)-**59** (Scheme 9).

### 2.7. Highly Regioselective Ring Opening of Epoxides using NaN_3_: A Short and Efficient Synthesis of (−)-Cytoxazone

**Scheme 10:**
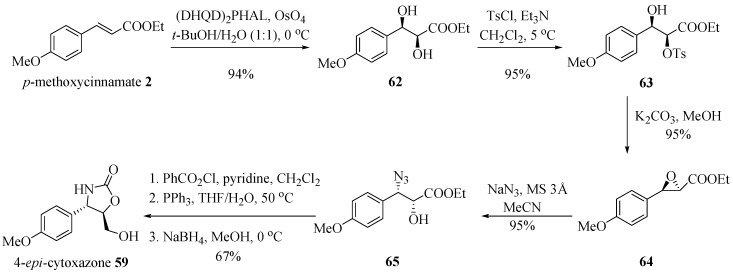
Total synthesis of (−)-cytoxazone proposed by Barua et al. [23].

Barua et al. [23] applied a regioselective ring opening of epoxide, using NaN_3_ and molecular sieve as the catalyst to obtain (−)-cytoxazone from *p*-methoxycinnamate (Scheme 10). Asymmetric dihydroxylation of *p*-methoxycinnamate **2** by treatment with 1,4-bis(9-*O*-dihydroquinidine)-phthalazine [(DHQD) 2PHAL] under the so-called AD-mix-α^®^ conditions [24], generated diol **62** in 94% yield and 99% *ee*. This diol was treated with *p*-toluenesulfonyl chloride and triethylamine, in turn leading to the monotosylated product **63**, which in the presence of K_2_CO_3_ enabled the formation of epoxide **64** with no occurrence of epimerization. The regioselective azidolysis of **64** with NaN_3_ led to the azido alcohol **65**, which was converted into *epi*-cytoxazone **59** in 57% overall yield from **62**, following the protocol described by Nakata et al. (Scheme 1). *NOTE*: The synthetic strategy shown in Scheme 10 does not lead to the natural product structure (−)-(4*R*,5*R*)-cytoxazone, as indicated by the authors, but rather to the (4*S*,5*R*)-*epi*-cytoxazone (**59**), as corrected in the scheme below.

### 2.8. Stereoselective Synthesis of (−)-Cytoxazone and (+)-5-epi-Cytoxazone

**Scheme 11:**
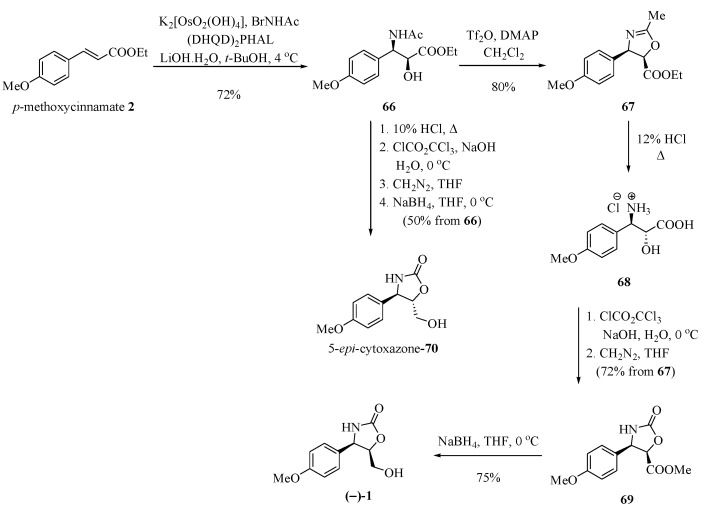
Total synthesis of (−)-cytoxazone by applying Sharpless asymmetric aminohydroxylation [25].

An asymmetric Sharpless aminohydroxylation reaction of *p*-methoxycinnamate with (DHQD)_2_PHAL produced amidoalcohol **66**, which was used to syntheze (−)-cytoxazone, by Saicic and his group [25] (Scheme 11). The *anti*-aminoalcohol configuration required for the synthesis of (−)-**1** was obtained by means of a configurational inversion of the amidoalcohol through an oxazoline. Thus, **66** was treated with Tf_2_O and DMAP, producing the *cis*-oxazoline **67** in an 80% yield. Acid hydrolysis led to hydroxyaminoacid **68**, which was converted into the cyclic carbamate **69** upon treatment with diphosgene and esterification with diazomethane. Finally, the reduction of the ester function with sodium borohydride produced the optically pure (−)-cytoxazone. The 5-*epi*-cytoxazone (**70**) was also prepared from the common intermediate **66** without requiring the correction of stereochemistry at C-5 (Scheme 11).

### 2.9. Regioselective and Diastereoselective Amination with the use of Chlorosulfonyl Isocyanate: A Short and Efficient Synthesis of (−)-Cytoxazone

**Scheme 12:**
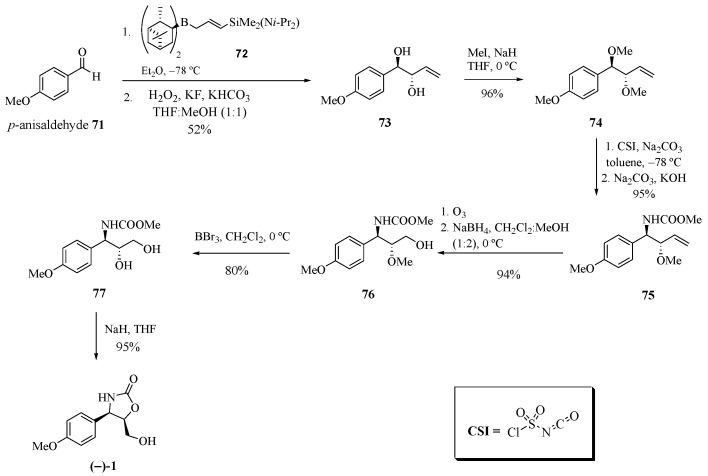
Total synthesis of (−)-cytoxazone from *p*-anisaldehyde [26].

Jung et al. [26] developed a new route of regio- and diastereoselective amination by applying chlorosulfonyl isocyanate (CSI) for the synthesis of (−)-cytoxazone (Scheme 12). The treatment of *p*-anisaldehyde (**71**) with *B*-[3-((diisopropylamino)dimethylsilyl)allyl] diisopinocamphenylborane (**72**) produced the *anti*-diol **73**. The dimethylation of compound **73** with NaH and iodomethane produced the *anti-*1,2-dimethyl ether **74**. The regio- and diastereoselective amidation using CSI in the presence of sodium carbonate and the reduction of the *N*-chlorosulfonyl group with aqueous sodium thiosulfate and sodium hydroxide produced the desired *anti*-amino ether **75** in a high diastereoselectivity (27:1). The ozonolysis of the double bond and the reduction of generated aldehyde led to alcohol ether **76**. Finally, demethylation with BBr_3_ and heterocyclization produced (−)-cytoxazone. 

### 2.10. Auxiliary Strategies to Prepare β-Amino Alcohols with Reductive Cross-coupling and the Synthesis of (−)-Cytoxazone

**Scheme 13:**
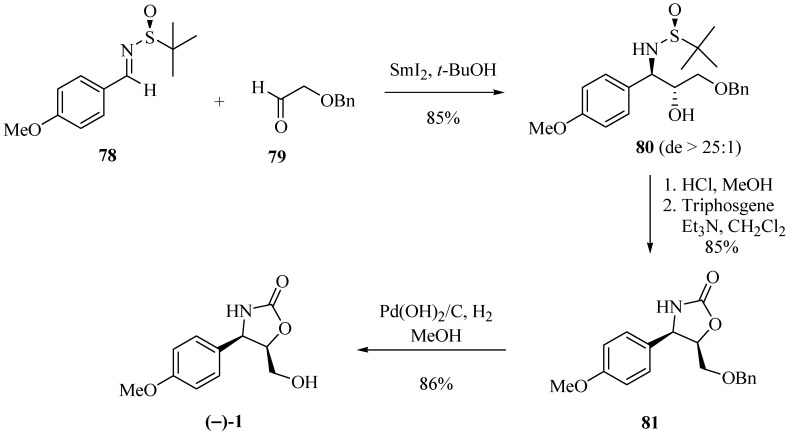
Total synthesis of (−)-cytoxazone using *N-tert*-butanesulfinyl imine as the chiral auxiliary [28].

Ellman et al. [27] first used *N-tert*-butanesulfinyl chiral imines providing good results in stereocontrolled alkylation reactions. Based on this chiral auxiliary, Bentley et al. [28], seeking to synthesize (−)-cytoxazone, obtained the *tert*-butanesulfinyil amine derivative **80** with good yield and high diastereoselectivity (>25:1) through a reductive SmI_2_-mediated cross-coupling reaction of the (*S*)-*N-tert*-butanesulfinyl imine **78** and aldehyde **79** (Scheme 13). It is important to note that the presence of the *p-*methoxyl substituent on the aromatic ring in **78** led to an increase in diastereoselectivity coupling. Removal under acidic conditions of the *tert*-butanesulfinyl (*t*-BS) auxiliary, heterocyclization, and debenzylation produced (−)-cytoxazone from **78** in 62% yield.

### 2.11. Synthesis of (−)-Cytoxazone and (+)-epi-Cytoxazone: The Chiral Pool Approach

**Scheme 14:**
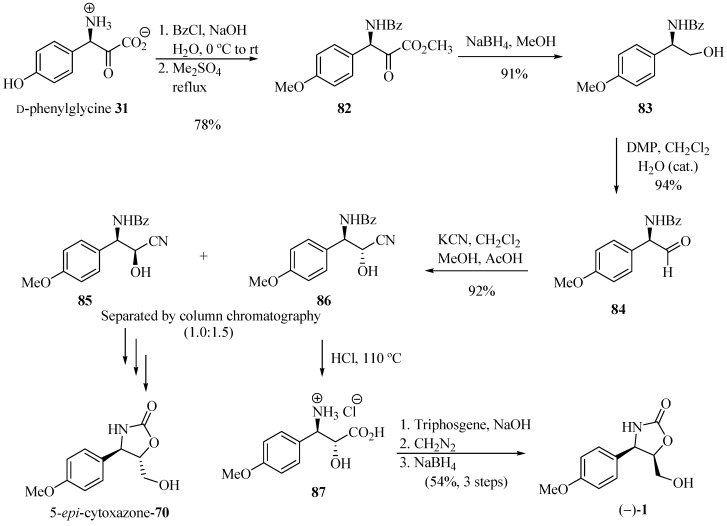
Total synthesis of (−)-cytoxazone and (+)-5-*epi*-cytoxazone from the (D)-(−)-4-hidroxyphenylglycine [29].

Saici et al. [29] reported the total synthesis of (−)-cytoxazone from *p-*hydroxy-D-phenylglycine **31** (Scheme 14). This amino acid was converted into its amino alcohol **83** after *N*-benzoylation, simultaneous esterification and methyl etherification, and the reduction of the ester group. Oxidation with water-catalyzed Dess-Martin periodinane led to aldehyde **84** in 94% yield and 98% *ee*, with no noticeable racemization. Homologation of the amino acid through the addition of KCN to the aldehyde **84** yielded a mixture of cyanohydrin diastereomers **85** and **86**. Separation of diastereomers by column chromatography produced the optically pure cyanohydrins, which were individually subjected to acid hydrolysis, cyclization, esterification, and reduction, leading to (−)-cytoxazone and (+)-5-*epi*-cytoxazone (**70**).

### 2.12. Short-step and Scalable Synthesis of (±)-Cytoxazone

**Scheme 15:**
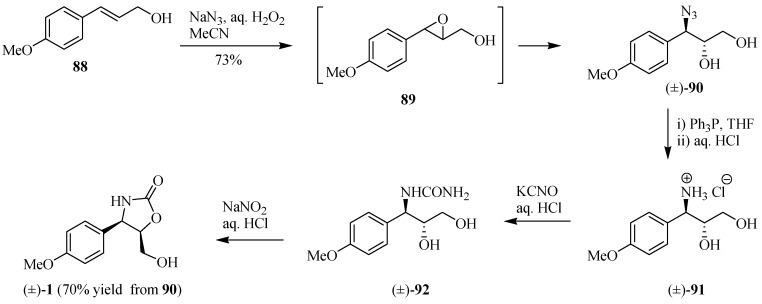
Synthesis of (±)-cytoxazone reported by Sugai and co-workers [30].

Sugai and co-workers [30] introduced a new one-pot azidohydroxylation procedure from **88** by combining the mixture of NaN_3_-H_2_O_2_-CH_3_CN to prepare (±)-cytoxazone by means of an epoxy-generated intermediate **89** (Scheme 15). The opening of epoxide **89** happened immediately after its formation through the presence of NaN_3_ in the medium reaction without any further addition of a Lewis acid to give azidoalcohol (±)-**90**. Reduction of the azide group led to amine salt **91**, after acidification with aqueous HCl, which was treatment with aqueous KCNO and an additional 0.2 eq. of aqueous HCl to produce *N*-carbamylamino alcohol (±)-**92**. Treatment with NaNO_2_ led to a nitrosation-deaminocyclization cascading sequence, yielding (±)-cytoxazone in 70% yield (three steps). Note that this strategy does not require any protection of the hydroxyl groups.

### 2.13. One-pot Sequential Catalytic Asymmetric Epoxidation-regioselective Epoxide-opening Process. Application for the Synthesis of (−)-Cytoxazone

**Scheme 16:**
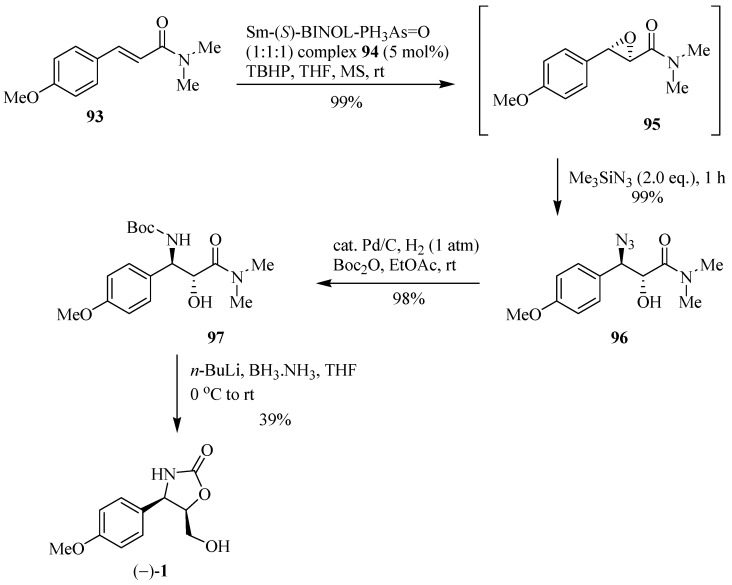
Synthesis of (±)-cytoxazone reported by Shibasaki and co-workers [31].

The application of a study executed by Shibasaki et al. [31] on the asymmetric epoxidation of α,β-unsaturated amide systems, in the presence of the Sm-(*S*)-BINOL-Ph_3_-As=O complex **94** and the immediate epoxide-opening process leading to the corresponding *anti*-β-azido-α-hydroxyamide, with complete regioselectivity and excellent *ee* (>99%) allows for the synthesis of (−)-cytoxazone (Scheme 16). Thus, the formation of the α,β-epoxy amide **95** and its immediate opening with Me_3_SiN_3_ provided efficient access to the β-azido alcohol **96**. Reduction of the azide group and subsequent *N*-Boc protection led to intermediate **97**. Finally, the reduction of the tertiary amide with *n*-BuLi and BH_3_.NH_3_ and immediate cyclization produced (−)-cytozaxone in 39% yield.

### 2.14. Enantioselective Synthesis of (−)-Cytoxazone and (+)-epi-Cytoxazone, Novel Cytokine Modulators via Sharpless Asymmetric Epoxidation and L-Proline Catalyzed Mannich Reaction

**Scheme 17:**
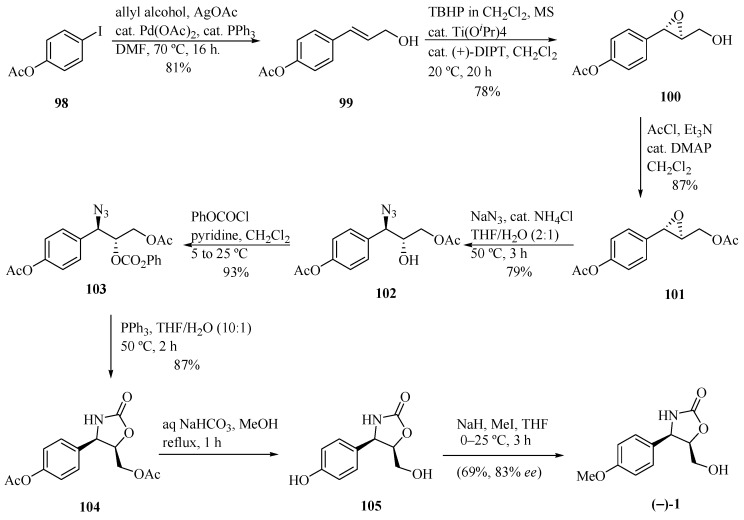
Total synthesis of (−)-cytoxazone reported by Sudalai and Paraskar [32].

Sudalai and Paraskar [32] developed interesting routes for the synthesis of (−)-cytoxazone and (+)-*epi*-cytoxazone by means of the Ti-catalyzed Sharpless asymmetric epoxidation of allylic alcohol and L-proline catalyzed Mannich reaction, respectively, as key steps to introducing the stereocenters into the molecule (Scheme 17). The Pd-catalyzed arylation of allylic alcohol with iodine **98** produced the *trans*-allylic alcohol **99**, which was converted into the chiral epoxide **100** via Sharpless asymmetric epoxidation [(+)-diisopropyl tartrate ((+)-DIPT), Ti(Pr*^i^*O)_4_, anhyd. *tert*-butyl hydroperoxide (TBHP)]. Acetylation of the hydroxyl led to epoxyacetate **101**. Regioselective opening, using NaN_3_ and NH_4_Cl (cat.), yielded the *trans*-azido alcohol **102**. Subsequently, successive reactions of alcohol protection, reductive cyclization using PPh_3_, basic hydrolysis of both acetate groups, and methylation led to (−)-cytoxazone in 56% yield from **102** and 83% *ee*.

**Scheme 18:**
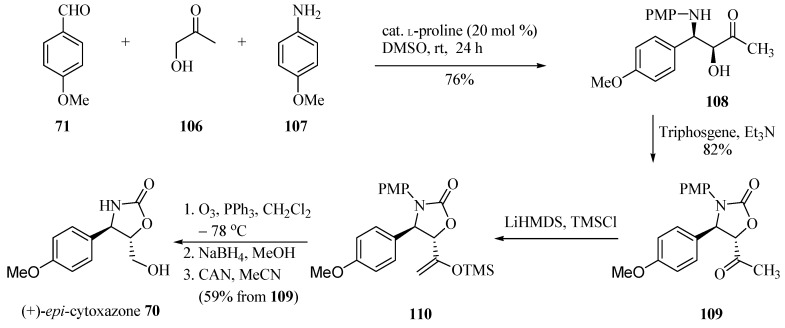
Total synthesis of (+)-*epi*-cytoxazone reported by Sudalai and Paraskar [32].

By contrast, the same authors [32] proposed another approach for the synthesis of (+)-*epi*-cytoxazone (**70**, Scheme 18). Thus, 4-methoxybenzaldehyde (**71**) was condensed with hydroxyacetone (**106**) and *p*-anisidine (**107**) in the presence of L-proline (30 mol %) to prepare chiral amino alcohol **108** in 76% and a (2:1) *syn/anti* ratio. The diastereomers were separated by column chromatography. The cyclization with triphosgene generated oxazolidinone **109** in 82% yield. Finally, silyl enol ether **110** was prepared from **109** by treatment with LiHMDS and TMSCl, which was subjected to ozonolysis without further purification. The reduction of generated aldehyde and *N*-PMP deprotection with CAN yielded (+)-*epi*-cytoxazone (**70**) in 59% yield from **109** and 81% *ee*.

### 2.15. A Concise Synthesis of (−)-Cytoxazone and (−)-4-epi-Cytoxazone using Chlorosulfonyl Isocyanate

Jung et al. [33] reported on the synthesis of (−)-cytoxazone and (−)-4-*epi*-cytoxazone from *p*-anisaldehyde (**71**) conducted in six steps, resulting in good diastereoselectivity. The key step in the synthesis includes the regio- and diastereoselective amination of the *anti*- and *syn*-1,2-dimethyl ethers with chlorosulfonyl isocyanate and the subsequent regioselective cyclization of the diols to build of the oxazolidin-2-one cores. This strategy proved to be equal to that previously published by the same authors in 2005 (see Scheme 12).

### 2.16. Diastereoselective and Enantioselective Henry (Nitroaldol) Reaction using a Guanidine-thiourea Bifunctional Organocatalyst. Application to the Synthesis of (4S,5R)-epi-Cytoxazone

**Scheme 19:**
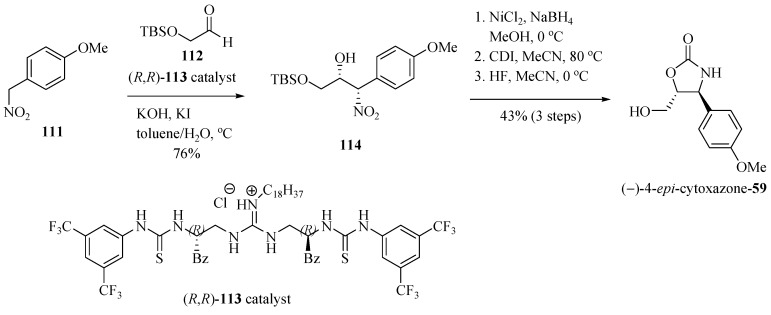
Synthesis of (4*S*,5*R*)-*epi*-cytoxazone (**59**) reported by Nagasawa et al. [34].

Nagasawa and his team [34] reported the application of highly diastereoselective and enantioselective Henry reaction (nitroadol), between the aldehyde **112** and 1-methoxy-4-nitromethylbenzene (**111**), using the bifunctional guanidine-thiourea **113** as a chiral catalyst, producing 1,2-*syn*-nitro alcohol **114** used to prepare (−)-*epi*-cytoxazone (Scheme 19). The formation of the oxazolidinone ring came through a sequence of reduction of the nitro group, cyclization, and desilylation steps, which produced (−)-4-*epi*-cytoxazone (**59**) in 33% overall yield and 95% *ee* from **111**.

### 2.17. NaIO_4_-mediated Asymmetric Bromohydroxylation of α,β-Unsaturated Carboxamides with High Diastereoselectivity: A Short Route to (−)-Cytoxazone 

**Scheme 20:**
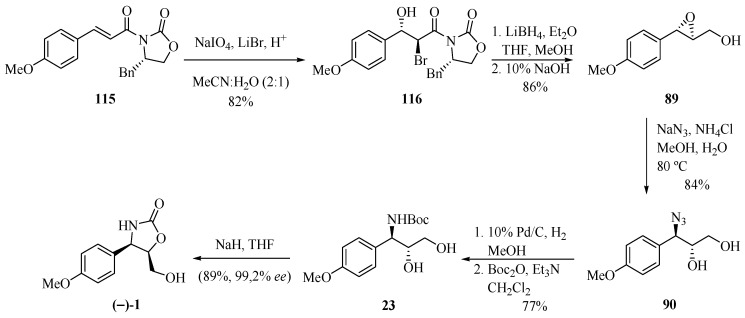
Total synthesis of (−)-cytoxazone proposed by Sudalai et al. [35].

Following the same line of research, Sudalai and co-workers [35] reported another contribution to the preparation of (−)-cytoxazone (Scheme 20), in addition to other previously published by the main author [32]. The diastereoselective bromohydroxylation reaction of the α,β-unsaturated carboxamide **115**, when in the presence of NaIO_4_ and LiBr, generated bromohydrin **116**. The chiral residue was removed when in presence of LiBH_4_, followed by treatment with 10% NaOH, in turn leading to epoxyalcohol **89**, whose regioselective opening with NaN_3_ yielded the respective azido alcohol **90**. The latter was converted into a product of interest after the reduction of the azide group, *N*-Boc protection, and cyclization.

### 2.18. Enantioselective Synthesis of (−)-Cytoxazone and (+)-epi-Cytoxazone via Rh-catalyzed Diastereoselective Oxidative C–H Aminations

**Scheme 21:**
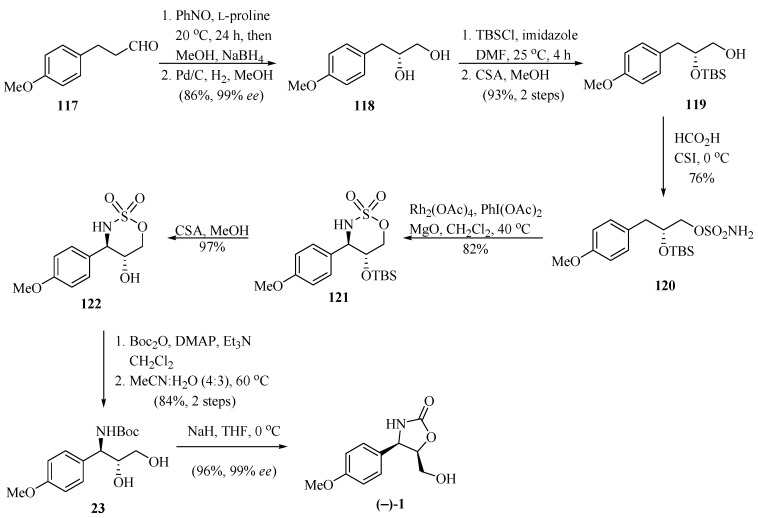
Synthetic approach of (−)-cytoxazone reported by Sudalai and his group [36].

In another study, following the same line of research on the construction of oxazolidinone rings, Sudalai and his group [36], reported on an elegant enantioselective synthetic route for the preparation of (−)-cytoxazone (Scheme 21). The reaction of aldehyde **117** with nitrosobenzene, as the oxygen source, in the presence of L-proline, followed by its reduction with NaBH_4_ led to the crude aminoxy alcohol. The subsequent reduction of the crude aminoxyalcohol with Pd/C and an atmosphere of H_2_ produced chiral diol **118** in 86% yield and 99% *ee*. The bis-TBS-protection and selective deprotection of the primary hydroxyl from **118** generated the corresponding silyl ether **119**. This alcohol was converted into the sulfamate ester **120** using known conditions (HCO_2_H and CSI). Selective γ-C-H insertion of the sulfamate ester **120** was carried out in the presence of a catalytic amount of Rh_2_(OAc)_4_ and PhI(OAc)_2_ as the oxidizing agent with *anti* (10:1) diastereoselectivity to produce the corresponding six-membered ring insertion product **121**. The *anti-*diastereomer **121** was readily separated by column chromatography. The sequence of desilylation, carbamoylation with Boc_2_O of the -NH moiety of oxathiazinane **122**, followed by ring opening of the crude *N*-Boc protected oxathiazinane with aqueous CH_3_CN and regioselective intramolecular cyclization, generated (−)-cytoxazone. The preparation of (+)-*epi*-cytoxazone was performed from aldehyde **117**, similarly to (−)-cytoxazone, by exchanging the L-proline for D-proline. It is important to mention that the second stereocenter in diol **118** can be generated with excellent distereoselectivity by Rh-catalyzed intramolecular amidation of the γ-C-H bonds of carbamate or sulfamate esters.

### 2.19. Iridium(I)-catalyzed Regio- and Enantioselective Allylic Amidation

**Scheme 22:**
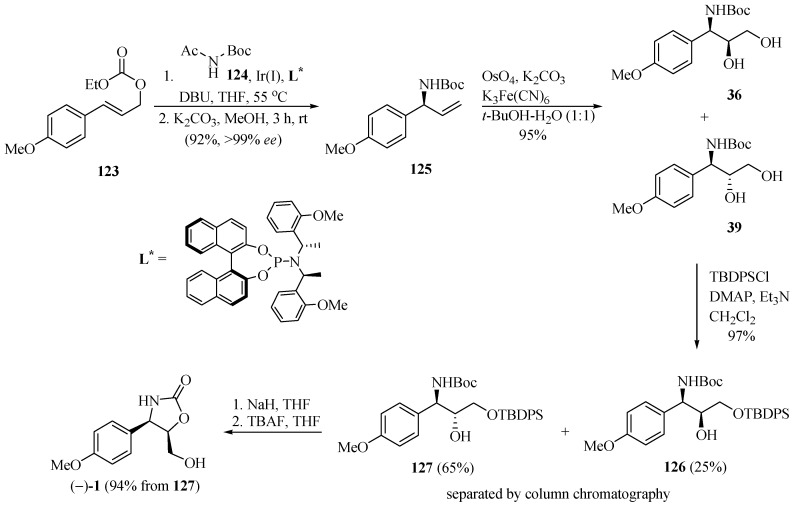
Total synthesis of (−)-cytoxazone reported by Han and Singh [37].

Han and Singh [37] developed a new route for the asymmetric synthesis of (−)-cytoxazone by applying an Ir(I)-catalyzed intermolecular allylic amidation reaction of aryl allyl carbonate with soft nitrogen nucleophiles and a chiral phosphoramidite ligand (L*) combined with the Sharpless dihydroxylation reaction (Scheme 22). The sequence of a one-pot process of Ir(I)-catalyzed allylic amidation of aryl allyl carbonate **123** with *N*-acetyl-*N*-*tert*-butyl carbamate **124** and chiral phosphoramidite ligand (L*), followed by *N*-deacetylation, led to the corresponding *N*-Boc protected allyl amine **125** in 99% *ee*. The Sharpless dihydroxylation reaction provided a mixture of *syn*-**36/***anti*-**39** diols in a 3:7 ratio. After converting the diols into their silylated ethers **126** and **127**, these were separated by column chromatography. Finally, the cyclization of **127**, performed in five steps starting from **123**, by treatment with NaH and desilylation, produced (−)-cytoxazone in 52% yield.

### 2.20. An Advance on Exploring N-tert-Butanesulfinyl Imines in Asymmetric Synthesis of Chiral Amines

**Scheme 23:**
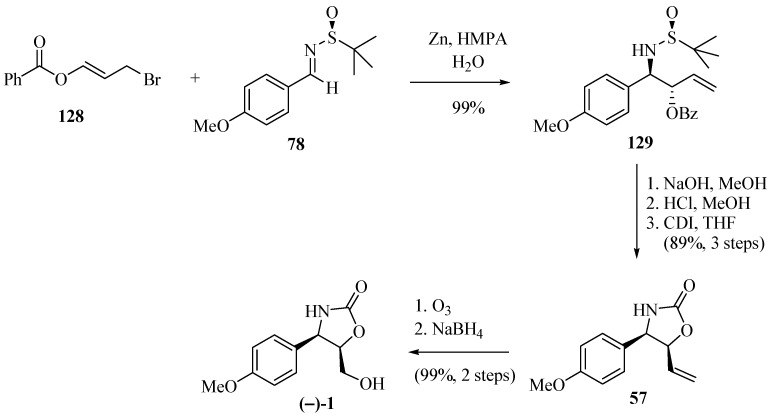
Synthesis of (−)-cytoxazone described by Lin and co-workers [38].

The application of the allylation of *N-tert*-butanesulfyl imine by the benzoyl-substituted allyl bromide **128** was illustrated by the synthesis of (−)-cytoxazone and described by Lin et al. [38] (Scheme 23). The allylation of imine **78** with **128** in the presence of Zn/HMPA produced **129** (99% yield, 96:4 *rd*). The routine removal of the benzoyl group and the chiral auxiliary, followed by ring closure in the presence of carbonyldiimidazole (CDI), produced **57**. The ozonolysis of the double bond in **57**, followed by the reduction of the generated aldehyde, completed the synthesis of the desired (−)-cytoxazone.

### 2.21. Asymmetric Synthesis of Chiral Amines by Highly Diastereoselective 1,2-Additions of Organometallic Reagents to N-tert-Butanesulfinyl Imines

**Scheme 24:**
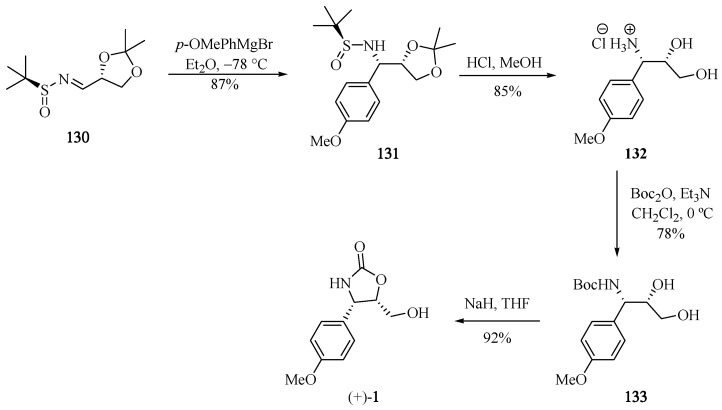
Total synthesis of (+)-cytoxazone reported by Babu and co-workers [39].

The synthetic strategy for the (+)-cytoxazone reported by Babu et al. [39] begins with the new chiral *N*-sulfinimine **130** (Scheme 24). The stereoselective 1,2-addition of the Grignard *p*-OMePhMgBr into **130** led to respective sulfonamide **131**, whose *t*-butylsulfonyl (*t*-BS) and 1,3-dimethyl acetal groups were removed in a single step in acidic media, producing the corresponding *syn*-β-aminoalcohol **132**. Finally, the *N*-Boc protection and heterocyclization produced (+)-cytoxazone in 53% overall yield from **130** (four steps).

## 3. Conclusions

This review illustrates the various creative methods used for the synthesis of (−)-cytoxazone (**1**) and its analogs from the first approaches to the present day. For example, in several synthetic approaches, cinnamic acid derivatives were used as substrates and α-hydroxy-β-amino functions were introduced in a crucial step by enantioselective Sharpless epoxidation, dihydroxylation, and aminohydroxylation, or by using certain well-known chiral inducers (Evans’s oxazolidinones, *N*-*tert*-butanesulfinyl imines), as well as by other less well-known inducers. The large number of synthetic routes to this compound and its congeners evince the various methods used by chemists to prepare this class of compounds, stimulated by its relatively simple structure in addition to its biological importance as a potent cytokine modulator and its possible use as an immunotherapeutic agent.
